# A Crp-Dependent Two-Component System Regulates Nitrate and Nitrite Respiration in *Shewanella oneidensis*

**DOI:** 10.1371/journal.pone.0051643

**Published:** 2012-12-11

**Authors:** Yangyang Dong, Jixuan Wang, Huihui Fu, Guangqi Zhou, Miaomiao Shi, Haichun Gao

**Affiliations:** Institute of Microbiology and College of Life Sciences, Zhejiang University, Hangzhou, Zhejiang, China; University of Glasgow, United Kingdom

## Abstract

We have previously illustrated the nitrate/nitrite respiratory pathway of *Shewanella oneidensis*, which is renowned for its remarkable versatility in respiration. Here we investigated the systems regulating the pathway with a reliable approach which enables characterization of mutants impaired in nitrate/nitrite respiration by guaranteeing biomass. The *S. oneidensis* genome encodes an *Escherichia coli* NarQ/NarX homolog SO3981 and two *E. coli* NarP/NarL homologs SO1860 and SO3982. Results of physiological characterization and mutational analyses demonstrated that *S. oneidensis* possesses a single two-component system (TCS) for regulation of nitrate/nitrite respiration, consisting of the sensor kinase SO3981(NarQ) and the response regulator SO3982(NarP). The TCS directly controls the transcription of *nap* and *nrfA* (genes encoding nitrate and nitrite reductases, respectively) but regulates the former less tightly than the latter. Additionally, phosphorylation at residue 57 of SO3982 is essential for its DNA-binding capacity. At the global control level, Crp is found to regulate expression of *narQP* as well as *nap* and *nrfA*. In contrast to NarP-NarQ, Crp is more essential for *nap* rather than *nrfA*.

## Introduction

*Shewanella oneidensis*, a Gram-negative γ-proteobacterium, has received enormous attention for its potential applications in bioremediation of heavy metals and energy generation via fuel cells [1]. In addition to utilizing oxygen as a terminal electron acceptor (EA), the organism can anaerobically respire on various organic and inorganic substrates, including fumarate, nitrate, nitrite, thiosulfate, trimethylamine *N*-oxide (TMAO), dimethylsulfoxide (DMSO), Fe(III), Mn(III) and (IV), Cr(VI), and U(VI). In order to reduce these diverse EAs, the *S. oneidensis* genome encodes different respiratory systems [2]. Over the last two decades, many of these anaerobic pathways have been elucidated, especially those involved in the reduction of toxic metals [1]. A common feature is that tetraheme cytochrome *c* CymA is shared by many of these branched respiratory pathways. This protein, located in the cytoplasmic membrane with a large portion exposed to the periplasm, is a key player in electron transfer between quinols and its redox partners [2]. In contrast, terminal reductases, such as *fccA* (SO0970), *dmsA* (SO1429), *napA* (SO0848), *nrfA* (SO3980), and *torA* (SO1232) encode fumarate, DMSO, nitrate, nitrite, and TMAO reductases respectively, are generally substrate-specific [3]–[7].

Most *Shewanella* genomes encode two isoforms of periplasmic nitrate reductase (NAP), encoded by *napEDABC* (*nap-α*) and *napDAGHB* (*nap-β*). The latter lacks *napC*, whose product is the essential and dedicated electron transport protein for NapAB [8]–[9]. Consequently, the *nap-α* operon alone is able to catalyze the reduction of nitrate to nitrite, whereas in the absence of *nap-α*, as in *S. oneidensis* [3]–[4], [9]–[10], the *nap-β* operon requires *cymA*. In the case of periplasmic nitrite reductase (NRF), *nrfA*-containing organisms usually possess either NrfAH or NrfABCD, in which NrfH or the complex of NrfBCD delivers electrons to NrfA [11]. Surprisingly, only *nrfA* has been identified in all sequenced *Shewanella* except for *S. denitrificans*. Similar to NAP-β, NrfA recruits CymA for electron transportation from the menaquinone pool [4]. One consequence of sharing CymA is that reduction of nitrite to ammonium does not start until nitrate is exhausted completely [4]. This is achieved via NapB, the small subunit of nitrate reductase complex, which diverts electrons from CymA to NapA exclusively when nitrate is present. Removal of NapB enables simultaneous nitrate and nitrite reduction, resulting in a characteristic pseudo-one-step reduction of nitrate [4].

Regulation of nitrate/nitrite reduction has been extensively studied in the model organism *Escherichia coli* [12]–[19]. Transcriptional control of this process has been mainly accredited to three systems, Fnr (*f*umarate *n*itrate reductase *r*egulator), two-component systems (TCS) NarX*_Ec_*-NarL*_Ec_*, and NarQ*_Ec_*-NarP*_Ec_*. While Fnr is the master regulator responsible for the major changes caused by aerobic to anaerobic growth switch, it directly activates operons encoding nitrate/nitrite reductases [15], [20]–[24]. Homologous TCS NarX*_Ec_*-NarL*_Ec_* and NarQ*_Ec_*-NarP*_Ec_*, distinguished from each other by a cysteine cluster found in NarX*_Ec_* only, have a relatively more specific role in regulation of nitrate/nitrite reduction [15], [25]. Almost all of operons for nitrate/nitrite reductases are directly controlled by these two systems. While NarX*_Ec_*-NarL*_Ec_* and NarQ*_Ec_*-NarP*_Ec_* function concertedly in some cases (e.g. *nirBDC*) [26], they are mostly antagonizing against each other by binding to the same or different sites (e.g. *napFDAGHBC*) [14]–[15], [19]. Furthermore, these two systems cross-regulate each other in an asymmetric manner [19], [27].

Although little is known about the regulation of nitrate and nitrite respiration in *S. oneidensis*, it is clearly different from the *E. coli* paradigm. On one hand, Fnr (previously EtrA), in contrast to its *E. coli* counterpart, acts as a fine-tuning regulator of respiration of a number of EAs, including nitrate [28]–[29]. On the other hand, Crp (*c*yclic AMP *r*eceptor *p*rotein) plays an essential role in regulating anaerobic respiration evidenced by the observation that a *crp* null mutant is deficient in anaerobic respiration of nitrate, Fe(III), Mn(IV), fumarate, and DMSO [30]. The regulatory mechanism of *S. oneidensis* Crp appears to be the same as the canonical cAMP-Crp system because adenylate cyclases CyaA and CyaC are found to be required for Crp activation [31]. Additionally, NarQ (SO3981) is the only protein that has been annotated as a component of the Nar regulatory system in *S. oneidensis* [2]. Previously we have unveiled the nitrate/nitrite respiration pathway in *S. oneidensis* and found a number of unique features, warranting further investigation into its regulatory systems [4]. Here, we present evidence to suggest that nitrate/nitrite respiration is under the direct control of a TCS system, which in turn is governed by global regulator Crp.

## Results

### Cultivation Conditions Suitable for Characterizing *S. Oneidensis* Mutants Defective in nitrate/nitrite Respiration

Since the elucidation of the anaerobic nitrate and nitrite respiration in *S. oneidensis* [4], we have taken on exploration of the regulatory proteins mediating the process. However, such an investigation was hampered by extremely poor growth of the bacterium on nitrate and nitrite under anaerobic conditions. In liquid culture, an EA concentration of 2 mM is insufficient to warrant quantitative assessment of growth differences of *S. oneidensis* [28]. Due to the toxicity of nitrite, nitrate and nitrite as the sole EA are routinely supplemented at concentrations no more than 5 mM, resulting in the extremely low cell densities (<0.1 of OD_600_) [3]–[4]. Mutants lacking one of the genes involved in nitrate/nitrite respiration or regulation become uncharacterizable because of further reduction of biomass. These obstacles prompted us to test for cultivation conditions that allow occurrence of nitrate/nitrite respiration and a biomass enough for examination of such mutants.

**Figure 1:**
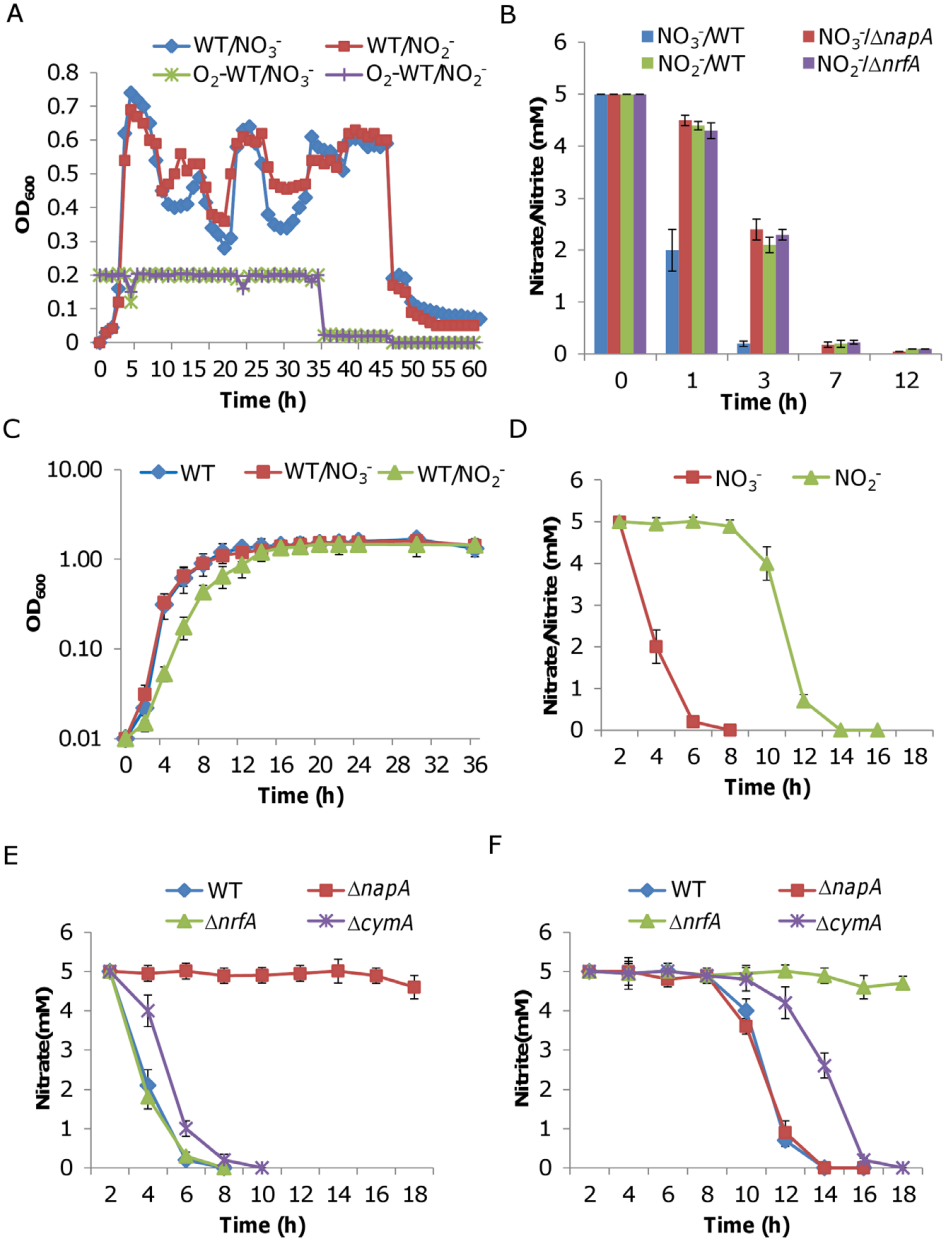
Growth and nitrate/nitrite reduction of *S. oneidensis* strains. **A.** Growth of *S. oneidensis* wild type (WT) in a chemostat supplemented with 5 mM NaNO_3_ (WT/NO_3_^−^) or NaNO_2_ (WT/NO_2_^−^) upon inoculation. Oxygen levels (O_2_-WT/NO_3_^−^ and O_2_-WT/NO_2_^−^) were set at 20% initially, reduced to 2% and 0% at 36 and 48 h after inoculation, respectively. Similar results were obtained from Δ*napA* and Δ*nrfA*, which are unable to respire on nitrate and nitrite respectively (not shown for clarity). **B.** Nitrate/nitrite concentrations in the chemostat cultures shown in A. Nitrate or nitrite was added 3, 15, 27, 39 h after the inoculation during growth and assayed at 1, 3, 7, 12 h after each addition using IC. Experiments were repeated with Δ*napA* or Δ*nrfA* for comparison. NO_3_^−^/WT and NO_3_^−^/Δ*napA* represent the wild type and Δ*napA* cultures in the presence of nitrate and NO_2_^−^/WT and NO_2_^−^/Δ*nrfA* represent the wild type and Δ*nrfA* cultures in the presence of nitrite. **C.** Growth of *S. oneidensis* in batch cultures under aerobic conditions. WT/NO_3_^−^, and WT/NO_2_^−^ represent growth with nitrate and nitrite, respectively. Growth in the absence of either chemical (WT) is included for comparison. **D.** Nitrate/nitrite respiration. Shown are concentrations in the batch cultures shown in C. **E.** Nitrate respiration. Shown are nitrate concentrations in the batch cultures with Δ*napA*, Δ*nrfA* or Δ*cymA*. **F.** Nitrite respiration. Shown are nitrite concentrations in the batch cultures with Δ*napA*, Δ*nrfA* or Δ*cymA*. Experiments were performed at least three times independently. Error bars represent the standard deviation (SD) (*n* = 3–6). In the case of chemostat, error bars are omitted for clarity.

*S. oneidensis* cells have been cultivated in shake flasks (uncontrolled batch), controlled batch bioreactors and chemostats for various research purposes and the effect of these cultivation methods on microbial physiology has been assessed recently [32]. As controlled cultivation technologies minimize culture heterogeneity through continual and thorough agitation and by monitoring and controlling all culture parameters, we first made an attempt to culture cells in a chemostat. *S. oneidensis*, were grown under carbon limitation at a constant dilution rate of 0.15 h^−1^ at various oxygen supply rates (Fig. 1A). The pH value was maintained at 7.0±0.05 and the temperature was set to 30°C. Lactate (20 mM) was used as the single carbon and energy source under aerobic and suboxic conditions, while nitrate or nitrite (5 mM) was used as the sole EA anaerobically. The oxygen levels in culture were set at 20%, 2% and 0% of air saturation for aerobic, suboxic and anaerobic conditions, respectively. Interestingly, oxygen at 20% and 2% levels did not introduce a significant change in cell densities, suggesting that *S. oneidensis* cells grow well under suboxic conditions (Fig. 1A). By contrast, removal of oxygen caused a drastic reduction in cell densities, especially in the case of nitrite with which the biomass was hardly visible when cells reached the steady state, resembling that observed in batch cultures under anaerobic conditions [3]–[4]. It is therefore conceivable that mutants with an impaired nitrite respiration capacity would not be able to grow under this tested condition. We then examined whether nitrate and nitrite respiration could occur with 20% or 2% oxygen. Nitrate and nitrite were added into chemostat cultures to a final concentration of 5 mM at points 3, 15, 27, 39 h after the inoculation and assayed in a time-course manner (1, 4, 6, and 12 h after the addition). With the exception that cells took a little longer to grow up in the presence of nitrite, neither of the chemicals showed significant impacts on growth in terms of growth rate and cell densities. As shown in Figure 1B, cells of the wild type strain respired nitrate after the addition evidenced by the appearance of nitrite in the cultures and that the faster decreasing rate of nitrate concentrations compared to that of Δ*napA*, which is unable to respire nitrate. In the case of nitrite, the reduction of concentrations in the wild type and Δ*nrfA* cultures was apparently due to the culture dilution rather than respiration because two strains displayed nearly identical rates, indicating that cells do not respire on nitrite. Moreover, this observation was independent of oxygen concentrations, rendering aerobic chemostat cultures unsuitable for studies on nitrite respiration of *S. oneidensis*.

We then tried with uncontrolled batch cultures. The experiment was carried out in a Bioscreen C growth monitoring instrument, which minimizes culture heterogeneity by plating all biological replicates in the same growth environment and measuring the optical density of the samples automatically [33], [34]. Growth of *S. oneidensis* in the presence of 5 mM nitrate or nitrite is shown in Figure 1C, while nitrate and nitrite concentrations are shown Figure 1D and Figure 1E, respectively. Unlike nitrate, nitrite at 5 mM reduced the growth rate significantly (approximately 25% by generation times), in agreement with its highly toxic feature. Surprisingly, the batch cultures were able to carry out reduction of both nitrate and nitrite. However, nitrite reduction is unlike nitrate reduction in that it could not commence until cells entered the stationary phase, indicating that the physiological status of cells is crucial to the process.

The batch cultures, unexpectedly, open a window for investigation of nitrate/nitrite respiration in *S. oneidensis*. To evaluate whether the culture conditions confer a sensitivity that enables differentiation of mutants with impaired nitrate/nitrite respiration, we repeated the experiment with Δ*cymA*, Δ*napA*, and Δ*nrfA* strains. All of these strains exhibited similar growth dynamics compared with the wild type in the presence of either chemical at 5 mM (Fig. S1A). Previously, we have shown that Δ*cymA* was completely deficient in nitrite reduction but retained a small nitrate reduction capacity when the characterization was performed under anaerobic conditions [4]. However, with batch cultures under aerobic conditions we found that the strain was able to carry out respiration of both nitrate and nitrite although at significantly reduced levels (Fig. 1E and 1F). In comparison, Δ*napA* and Δ*nrfA* totally lost the ability to respire nitrate and nitrite, respectively. Additionally, genetic complementation was performed and the observed defects of these mutants were rescued by a copy of the corresponding gene (Fig. S1B). These data, collectively, indicate that the batch cultures, although much more heterogeneous than those in controlled systems, are suitable for characterization of important components in nitrate/nitrite respiration.

### NapA is Responsive to Nitrate and NrfA is Responsive to Both Nitrate and Nitrite

**Figure 2:**
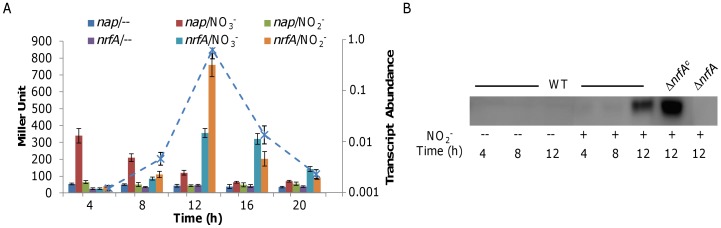
Expression analysis of *nrfA* during aerobic growth of *S. oneidensis*. **A.** A *lacZ*-based reporter analysis of the *nap* and *nrfA* promoters. Presented in columns is expression of *nap* and *nrfA* in the cells cultured aerobically in the absence of nitrite (/−) and in the presence of either nitrate (/NO_3_^−^) or nitrite (/NO_2_^−^). The *napA* mutant instead of the wild type was used here to keep nitrate unreduced. The *nrfA* mRNAs in the samples growing with NaNO_2_ were also analyzed by qRT-PCR and presented (dash line, abundance relative to 16 S rRNA). Error bars represent the standard deviation (SD) (*n* = 3). **B.** Western analysis of the cell samples used in A. Wild type cells grown in the absence or presence of nitrite at indicated time points were assayed, respectively. The complemented Δ*nrfA* (Δ*nrfA^c^*, carrying P*_nrfA_-nrfA*) exhibited over-production of NrfA and Δ*nrfA* was used as the negative control.

Discovery of the delay in nitrite reduction under aerobic conditions implicates novel features of the regulatory mechanisms controlling the process. As a first step to investigate, we examined transcription of *napA* and *nrfA* in a time-course manner using a *lacZ* reporter system and qRT-PCR [34], [35]. Transcriptional fusion vectors were constructed by placing ∼300 bp upstream sequences of the *nap* operon (P*_nap_*) and *nrfA* (P*_nrfA_*) before the full-length *E. coli lacZ* within pTP327 and introduced into Δ*napA*, in which conversion of nitrate to nitrite is prevented. Expression from P*_nap_* in both strains was found to be induced by nitrate but not nitrite anytime when they were added (Fig. 2A). On the contrary, in the presence of either nitrate or nitrite cells expressed β-galactosidase driven by P*_nrfA_* slightly before the stationary phase but robustly afterwards. Independent qRT-PCR analysis validated the observation (Fig. 2A), reinforced the view that NrfA is made in an abundant amount only when it is needed. To test whether the same scenario occurs at protein level, we raised an antibody against NrfA and used it to detect NrfA. Consistently, NrfA was present at an extremely low level before cells entered the stationary phase, when production of the protein increased dramatically (Fig. 2B).

### *S. Oneidensis* Possesses a NarP-NarQ System

Results presented thus far suggest that *S. oneidensis* is able to reduce nitrate anytime and nitrite at the time when cells are less respiratorily active. How does *S. oneidensis* sense these chemicals and mediate expression of the relevant genes for reduction? In *E. coli*, TCS NarP*_Ec_*-NarQ*_Ec_* and NarX*_Ec_*-NarL*_Ec_* directly mediate reduction of nitrate and nitrite. Both *narQ_Ec_* and *narP_Ec_* stand alone (belonging to different operons) while *narX_Ec_* and *narL_Ec_* form a single operon. A BLAST search using NarP*_Ec_*, NarQ*_Ec_*, NarX*_Ec_* and NarL*_Ec_* sequences against the *S. oneidensis* genome returned three significant hits that have at least a 90% sequence coverage and over 50% sequence similarity (Table 1). These proteins are SO1860, SO3981, and SO3982.

**Table 1:** Sequence similarities between *E. coli* NarXL*_Ec_*, NarQP*_Ec_* and *S. oneidensis* proteins.

	NarL*_Ec_*	NarX*_Ec_*	NarP*_Ec_*	NarQ*_Ec_*
SO3981 (NarQ)		62.70%		55.40%
SO3982 (NarP)	68.60%		72.10%	
SO1860	55.00%		56.90%	

**Figure 3:**
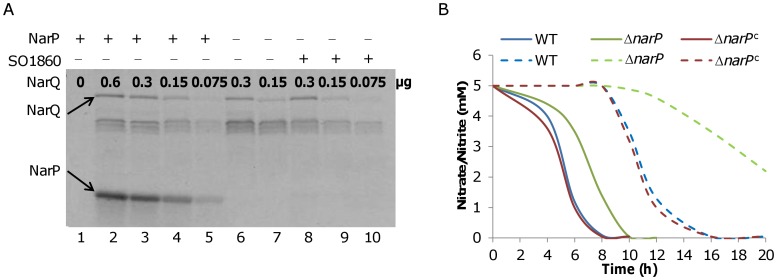
NarQ-NarP two-component system in aerobic respiration of nitrate/nitrite. A. Trans-phosphorylation of *S. oneidensis* NarP and SO1860 by NarQ^51–585^. The trans-phosphorylation assay was performed in the presence of [γ-^33^P]ATP with NarQ^51–585^ and 3 µg NarP, 3 µg SO1860, or without either NarP or SO1860. The resulting protein mixture was then resolved on an SDS-PAGE gel and the phosphorylated proteins were visualized by autoradiography. The numbers in the figure are the amount of NarQ^51–585^ protein used, in µg. Arrows indicate the position of phosphorylated NarQ^51–585^ or NarP protein. **B.** Nitrate/nitrite reduction in Δ*narP* under aerobic conditions. Cells of tested strains grown aerobically in the presence of nitrate or nitrate were collected at the indicated times. Concentrations of nitrate and nitrite (8 h and after) remaining in cultures were measured. *narP^c^* represents Δ*narP* complemented by a copy of *narP* on pHG101. The negative control (Δ*napA* and Δ*nrfA*) and error bars (SD, *n* = 3) were omitted for clarity.

SO3981 has been annotated to be NarQ as it lacks the signature of NarX*_Ec_* - the cysteine cluster [15]. *S. oneidensis narQ* is predicted to be co-transcribed with *SO3982*, implicating that SO3982 is the DNA-binding regulator for NarQ. However, NarQ may serve as a sensory kinase for more than one response regulators as its *E. coli* counterpart. If so, SO1860 is likely another partner of NarQ, although it has been shown to be able to receive a phosphoryl group from an orphan sensor kinase SO3457 [36]. To test whether these proteins function together in regulation of nitrate/nitrite respiration, we performed the trans-phosphorylation assay. *S. oneidensis* NarP, SO1860, and NarQ^51–585^ were cloned into Gateway entry vector pDNOR221, transferred into a protein expression system to attach an N-terminal His-tag, and the His-tagged proteins were expressed in *E. coli* and purified, mostly from inclusion bodies as described previously [37]. Like SO1860 [36], NarP failed to phosphorylate itself when ATP was included (Fig. 3A, lane 1). The NarQ^51–585^, however, was able to undergo autophosphorylation in the presence of ATP (Fig. 3A, lanes 6–7). This autophosphorylated NarQ^51–585^ in turn was able to phosphorylate NarP (Fig. 3A, lanes 2–5) but not SO1860 (Fig. 3A, lanes 8–10). These data strongly suggest that NarP is likely to be the DNA-binding regulator for NarQ and SO1860 may not be part of this system.

To reinforce this view, we constructed in-frame deletion mutation strains for each individual gene and determined the *in vivo* role of these three genes in nitrate and nitrite respiration. Under anaerobic conditions, Δ*narP* was unable to display visible growth when cells were inoculated into media containing nitrate or nitrite ranging from 5 mM as the sole EA while Δ*SO1860* grew as well as the wild type (Fig. S1C). We then cultured these mutants under aerobic conditions with either nitrate or nitrite and assessed reduction of nitrate and nitrite. Mutations in Δ*narP* and Δ*SO1860* were not able to elicit any noticeable difference in normal aerobic growth (data not shown) in the presence or absence of 5 mM nitrate or nitrite but their impact on nitrate/nitrite reduction varied. Δ*narP* was slightly delayed in its ability to reduce nitrate, completing conversion of nitrate to nitrite within 10 hours. However, Δ*narP* was significantly reduced in its ability to reduce nitrite as more than 40% of the initial nitrite concentration remained 20 hours after inoculation (Fig. 3B) and approximately 20% was still present even after 40 hours. From this observation, along with data from the *in vitro* trans-phosphorylation assay, we concluded that *S. oneidensis* possesses a NarP-NarQ TCS involved in nitrate and nitrite reduction, which controls *nrfA* more tightly than *nap*.

### Phosphorylation of Asp^57^ is Essential for *S. Oneidensis* NarP Binding Activity

The overall sequence conservation of the *E. coli* and *S. oneidensis* NarP proteins as well as the conservation of Asp^57^ suggests that activation by phosphorylation may occur at this residue (Fig. S2). To determine this and whether specific phosphorylation is required for NarP DNA binding, a *S. oneidensis* NarP mutation protein in which Asp^57^ was replaced with asparagine (57^DN^) by site-directed mutagenesis was created, expressed and purified from *E. coli*. The binding characteristics of the NarP, NarP-P, NarP(D57N), and NarP(D57N)^#^ (treated by phosphorylation agents) proteins were tested using a radio-labeled *nrfA* promoter DNA probe, which has been shown to be able to bind to NarP [37].

**Figure 4:**
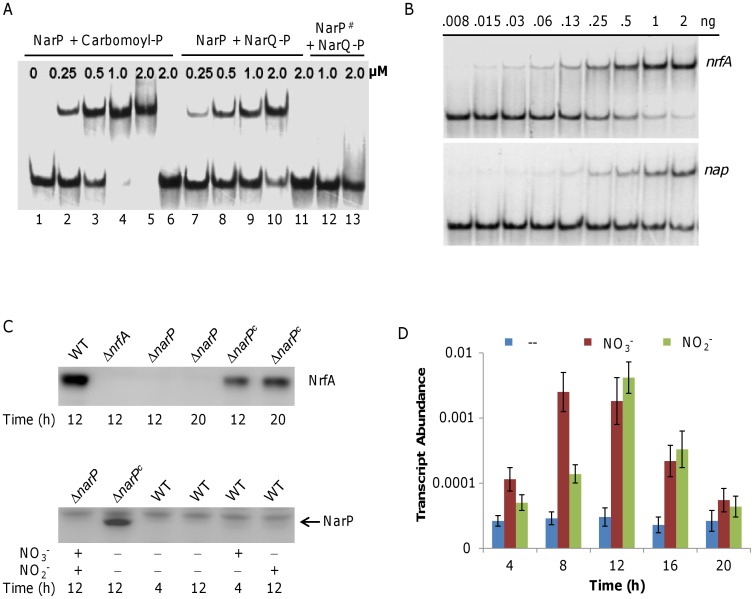
Binding analysis of NarP to *nap* and *nrfA* promoters. **A.** EMSA assay with carbamoyl phosphate (lanes 2–6) and NarQ^51–585^ (lanes 7–11) treated NarP. NarP^#^ represents NarP carrying a D57N mutation. Binding assays with NarP^#^ treated with NarQ^51–585^ and ATP are shown in lanes 12–13. All of the binding assays were performed with 2 ng of *nrfA* upstream fragments in the presence of 2 µg non-specific competitor DNA poly(dI**·**dC). Lanes 6 and 11 contain 10 µM of unlabeled *nrfA* upstream fragments as competitor DNA. The concentration of NarP or NarP^#^ is indicated in the figure (µM). **B.** The EMSA assay was performed with 2 µM phosphorylated NarP and various amounts of ^33^P end-labeled *nrfA* (−50 to −200 relative to the translation start codon) and *nap* (−50 to −200) upstream fragments. Non-specific competitor DNA, 2 µg poly(dI·dC), was added in all lanes. **C.** Western blotting analysis. Upper panel, analysis of NrfA in Δ*narP*. Cells grown in the presence of nitrite at the indicated time points were assayed. Lower panel, analysis of NarP. Cells grown in the presence of nitrate and/or nitrite at the indicated time points were assayed. In both panels, Δ*narP^c^* represents Δ*narP* containing pHG102-*narP* (P*_arcA_-narP*), in which *narP* is over-expressed. **D.** qRT-PCR analysis of the *narQ-narP* operon. The wild type cells grown with nitrate or nitrate aerobically were collected at the indicated time points and assayed. Abundance is given relative to 16S rRNA. Error bars represent the standard deviation (SD) (*n* = 3).

In contrast to the non-phosphorylated NarP which at the concentration of up to 4 µM could not bind to the *nrfA* promoter [37], significant binding to the *nrfA* promoter DNA occurred at a concentration of less than 0.25 µM for NarP-P (Fig. 4A, lane 2). The binding of NarP-P to the target promoter was not reduced by addition of the nonspecific competitor poly(dI**·**dC) DNA (Fig. 4A, all lanes), but was outcompeted by excess unlabeled probe (Fig. 4A, lane 6). These results demonstrate that phosphorylated NarP binds the *nrfA* promoter in a sequence specific fashion. Moreover, results showed that NarP treated by the phosphorylated NarQ^51–585^ yielded a similar binding pattern as by carbamoyl phosphate (Fig. 4A, lanes 7–10). On the contrary, neither NarP(D57N) nor NarP(D57N)^#^ proteins were able to bind the tested promoter DNA at concentrations up to 2 µM (Fig. 4A, lanes 12–13). All these data indicate that the residue 57 of NarP is the phosphorylation site and such phosphorylation is essential for the binding activity of NarP.

### The NarP-NarQ System Controls NRF More Tightly than NAP

Evidence based on the mutation analysis of NarP illustrates that this TCS is involved in respiration of nitrate and nitrite in *S.* *oneidensis*, and more importantly, imposes significantly different impacts on nitrate and nitrite reduction. We reasoned that this may be reflected by the binding characteristics of phosphorylated NarP to *nap* and *nrfA* promoter regions. We therefore performed an electrophoretic motility shift assay (EMSA) to directly evaluate binding of NarP on *nap* and *nrfA* upstream sequences with NarQ^51–585^ as the phosphor donor. The results showed that the DNA fragment covering the *nrfA* promoter bound to NarP phosphorylated by the NarQ^51–585^ protein efficiently (Fig. 4B, upper panel), while the *nap* promoter region exhibited a much lower binding capacity (Fig. 4B, lower panel). Independent confirmation came from the analysis of P*_nap_* and P*_nrfA_* in Δ*narP* grown to different stages with nitrate or nitrite. The β-galactosidase expression driven by P*_nap_* showed a relatively mild decrease (60% remaining) in Δ*narP* compared to the wild type. In contrast, the *lacZ* expression driven by P*_nrfA_* was nearly abolished (less than 10% remaining) (Fig. S3A). To examine the amount of NrfA proteins in Δ*narP*, we performed western blotting using anti-NrfA antibodies on the same samples. While we were able to detect NrfA in the wild type and complemented strains, we did not observe any detectable levels in the *narP* mutant (Fig. 4C, upper panel).

As a TCS system, it is conceivable that NarP is likely inducible by nitrate and/or nitrite. To examine this, we carried out an immunoblotting assay with crude protein extracts prepared from the wild type grown under various conditions using polyclonal antisera raised against *S. oneidensis* NarP. The amount of NarP in the wild type was too low to be confidently detected even in the presence of nitrate and nitrite. However, with the help of over-expressed NarP, we managed to observe that NarP was subjected to induction by both nitrate and nitrite (Fig. 4C, lower panel). To confirm this, transcription of the *narQ-narP* operon was assayed using qRT-PCR. Consistent with the Western blotting data, the transcription of the operon was rather low (Fig. 4D). Nevertheless, the results showed that up to 6 times more transcripts were produced in the presence of either nitrate or nitrite, solidifying that both chemicals strongly induce expression of *narQ-narP*.

### Crp Activates Expression of NarP-NarQ as well as NRF and NAP Directly

In *S. oneidensis*, three global regulators, ArcA, Crp, and Fnr have been shown to be involved in the regulation of respiration. While ArcA appears to be important in both aerobic and anaerobic respiration, the protein is unlikely to be involved in regulation of nitrate or nitrite respiration due to the lack of its binding sites in the upstream regions of the *nap*, *nrfA*, and *narQP* operons [34], [38]–[39]. In contrast, several lines of evidence indicates that Crp and Fnr mediate nitrate/nitrite reduction as well as Fnr- and Crp-binding sites have been identified in the control sequences of the *nap*, *nrfA*, and *narQP* operons, suggesting regulation in a direct manner [29]–[31], [34], [39].

**Figure 5:**
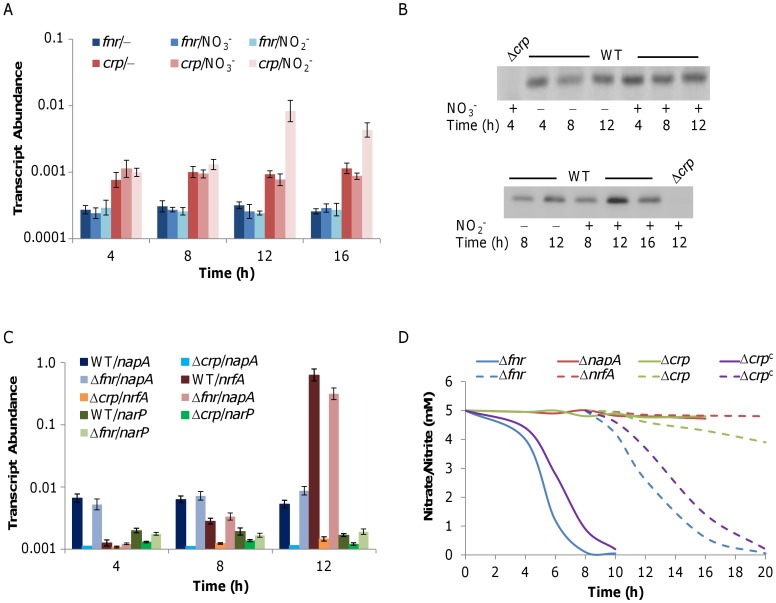
Crp and Fnr in aerobic nitrate/nitrite respiration of *S. oneidensis*. **A.** qRT-PCR analysis of *crp* and *fnr*. The wild type cells grown in the presence or absence of nitrate or nitrate aerobically were collected at the indicated time points and assayed. Expression level of each gene was presented under three conditions: –, no addition of nitrate or nitrite, NO_3_^−^, nitrate added, and NO_2_^−^, nitrite added. Abundance is given relative to 16 S rRNA. **B.** Western blotting analysis of Crp. Upper panel, the wild type cells cultured in the absence of nitrate or presence of nitrate at 4, 8, and 12 h were assayed. Lower panel, the wild type cells cultured in the absence of nitrite or presence of nitrite at 8, 12, and 16 h were assayed. Δ*crp* was used as the negative control. **C.** qRT-PCR analysis of *nap*, *nrfA*, and *narP* in cells grown with nitrate. The wild type, Δ*crp*, and Δ*fnr* mutant strains were assayed at indicated time points. Abundance is given relative to 16 S rRNA. **D.** Nitrate/nitrite assay. Cells of tested strains grown aerobically in the presence of nitrate or nitrate were collected at the indicated times. Concentrations of nitrate and nitrite (8 h and after) remaining in cultures were measured. Δ*crp^c^* represented the mutant containing a copy of *crp* on the complementation plasmid. Solid and dash lines represent cells grown in the presence of nitrate and nitrite, respectively. The wild type and Δ*fnr* were indistinguishable from each other and thus data for the wild type and Error bars (SD, *n* = 4) were omitted for clarity.

We compared the wild type and Δ*arcA* in their ability to reduce nitrate/nitrite under aerobic conditions and found that they were indistinguishable from each other (data not shown), confirming that ArcA is irrelevant in regulation of nitrate/nitrite respiration. Previously, using a *lacZ* reporter system, we have shown that the expression of *crp* under anaerobic conditions is roughly two times higher than that under aerobic conditions, but *fnr* does not respond to oxygen at the transcriptional level [34]. The experiment was performed with TMAO as EA because Δ*crp* was defective in growing on other EAs under anaerobic conditions. In this study, we were able to examine the responsiveness of *crp* to nitrate and nitrite under aerobic conditions (Fig. 5A). Results showed that i) *fnr* was expressed constitutively under all tested conditions as observed before [34], ii) *crp* was transcribed in a considerable amount in cells growing aerobically and cells that were respiratorily inactive (at stationary phase) in the absence of any anaerobic EA, resembling Fnr of *E. coli* [40], and iii) *crp* was not transcriptionally affected by the addition of nitrate in aerobic growing cells, and increased its expression about 2-fold in cells that grew into the stationary with nitrite. In addition, Crp at the protein level examined by western blotting with antibodies against Crp agreed with the expression pattern observed in the transcriptional analysis (Fig. 5B).

Next, we examined the effect of Crp and Fnr on expression of *nap*, *nrfA*, and *narP*. The qRT-PCR results showed that expression pattern of these genes was not significantly altered by the removal of Fnr compared with the wild type (Fig. 5C). By contrast, the loss of Crp resulted in a complete shutdown of transcription of *nap*, and reduced expression of *nrfA* and *narP* to roughly 5% and 30%, respectively, when cells were grown in the presence of nitrate (Fig. 5C). The same held true for the presence of nitrite in general (Fig. S3B). To further confirm, the samples used for qRT-PCR were subjected to nitrate and nitrite reduction assays. Conversion of nitrate to nitrite by either Δ*crp* or Δ*napA* was not observed when nitrate was added from inoculation (Fig. 5D), establishing that Crp is essential for nitrate respiration. As the experiment was performed with abundant oxygen, these results provide direct evidence that Crp functions under aerobic conditions. However, although the *crp* mutant displayed a severe defect in nitrite reduction, it retained a small share of nitrite reducing capacity under tested conditions (Fig. 5D). On the contrary, the *fnr* mutant behaved as the wild type under all tested conditions. In sum, the defect in nitrate/nitrite reduction introduced by the removal of Crp was much more severe than Δ*narP*, indicating that Crp not only regulates expression of *narQ*-*narP*, but also controls expression of *nap* and *nrfA* directly. It is worth noting that a copy of *crp* expressed *in trans* fails to fully restore the wild type capacity in either nitrate or nitrite respiration. As the complementation vector pHG101 has been shown here and before to cause over-expression of the cloned genes [35], the result implicates that Crp may function in a dose-dependent manner. The precise mechanism is currently under investigation.

## Discussion

In *S. oneidensis*, the nitrate/nitrite respiration pathway carries a few novel features [4]. First of all, neither nitrate nor nitrite enzymatic system is complete, missing otherwise essential NapC or NrfH, respectively. Instead, CymA delivers electrons to both nitrate and nitrite reductases NapA and NrfA. Moreover, NapB, the unessential small subunit of the nitrate reductase, preferentially transfers electrons from CymA to NapA. At the level of regulation, the novelties have also been revealed. For instance, Crp rather than Fnr is crucial in respiration of both nitrate and nitrite [30]. While investigation of the regulatory systems controlling the bacterial nitrate and nitrite respiration is undoubtedly appealing, it is hindered by the fact that mutants partially defective in the process do not afford a biomass for genetic analysis. Recently, it has been shown in *S. oneidensis* that a number of anaerobic respiratory systems can be synthesized in the presence of oxygen, although to a much lesser extent compared to that in the absence of oxygen [41]. This suggests that some of these systems may be functional in the presence of oxygen. Indeed, our results demonstrated that both nitrate and nitrite can be respired in agitated aerobic batch cultures despite the delay in the nitrite respiration. Such a cultivation condition, minimizing the inhibitory effect of nitrite, not only allows reaching the biomass required for biochemical and genetic analyses but also confers sensitivity high enough for differentiating the degree of defectiveness of mutants. With batch cultures under aerobic conditions, here we observed that neither Crp nor CymA was absolutely essential for synthesis of the nitrite terminal reductase in contrast to previous reports although loss of Crp shuts down the nitrate reductase completely [4], [30]. These data therefore justify re-evaluating the essential role of Crp on the respiration of other EAs.

**Figure 6:**
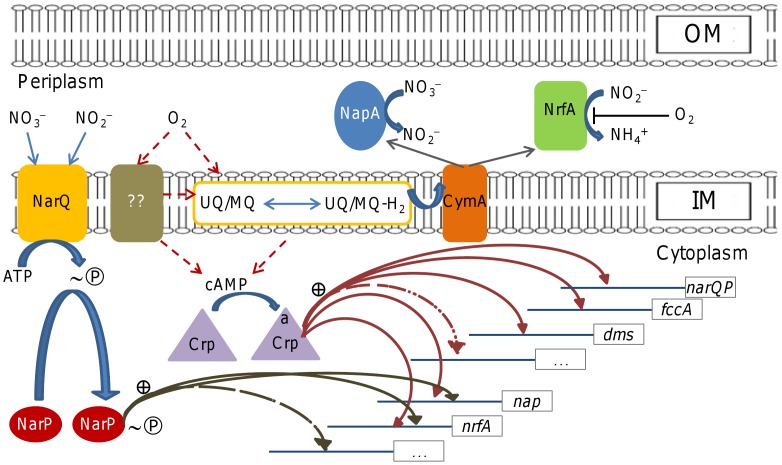
Model for regulation of nitrate/nitrite respiration in *S. oneidensis*. Shown is schematic diagram of regulation of nitrate/nitrite respiration. NarQ responds to both nitrate and nitrite and subsequently phosphorylates NarP, which activates *nap*, *nrfA*, and possibly more operons. Crp responds to cellular cAMP levels, whose fluctuation may be affected by an unknown oxygen-sensing protein and/or the redox states of quinol pools. The activated Crp proteins (labeled with a) bind to target genes to activate their transcription. The unconfirmed signal transduction pathways are shown in dash lines.

A major difference in regulatory systems between *S. oneidensis* and *E. coli* is that the former appears to utilize a single TCS to control nitrate and nitrite respiration (Fig. 6). In *E. coli*, homologous TCS NarX*_Ec_*-NarL*_Ec_* and NarQ*_Ec_*-NarP*_Ec_*, distinguished from each other by a cysteine cluster found in NarX*_Ec_* only, cross-function asymmetrically in regulation of nitrate and nitrite respiration [15], [19], [25], [27]. NarX*_Ec_* is preferentially stimulated by nitrate and exhibits a significant preference for phosphotransfer to NarL*_Ec_* whereas NarQ*_Ec_* is stimulated by both nitrite and nitrate and activates both NarP*_Ec_* and NarL*_Ec_* [19], [27]. In *S. oneidensis*, NarP but not SO1860, both of which are homologues to NarP*_Ec_* and NarL*_Ec_*, partners with NarQ (lacking the cysteine cluster) despite cross-talk between these TCSs in *E. coli*. As a single system, the *S. oneidensis* NarP-NarQ TCS is stimulated by both nitrate and nitrite but controls *nrfA* substantially more tightly than *nap* (Fig. 6).

The essentiality of global regulator Crp in *S. oneidensis* nitrate/nitrite respiration has been reported for nearly a decade [30]. In this study, we provided evidence that the *crp* mutant retained a small share of capacity in nitrite respiration. Nevertheless, the protein plays a predominant role in regulation of nitrate/nitrite reduction in *S. oneidensis* [30], [34]. It has been proposed that Crp directly regulates expression of most components of anaerobic respiratory systems given that predicted Crp-binding motifs are identified in upstream regions of their coding genes, including *narQP*, *nap* operon, *nrfA*, *dms* operon, and *cymA* [34]. Here, we demonstrated that all of *narQP*, *nap* operon, and *nrfA* require Crp for full expression, supporting the notion that the protein controls both regulation and synthesis of respiratory pathways. This scenario resembles *E. coli* Fnr [20]–[24]. In *E. coli*, Fnr is the predominant regulator that responds to O_2_ decrease and regulates by site-specific DNA-binding [42]. Our results show that the influence of *S. oneidensis* Fnr on nitrate/nitrite reduction under aerobic conditions was insignificant despite the presence of the predicted Fnr-binding sites in the upstream regions of the *nap*, *nrfA*, and *narQP* operons [9], [34]. Although the observation is arguably due to the establishment in *E. coli* that Fnr is not active under aerobic conditions, it agrees with multiple lines of evidence, most of which come from studies under anaerobic conditions [28]–[29], [34]. It is also natural to propose that Crp and Fnr cooperate with each other in regulation of nitrate/nitrite respiration as established in *E. coli* [39]. However, this Crp-Fnr synergy must be extremely limited in function because the mutant devoid of both *crp* and *fnr* behaves the same as the *crp* single mutant in respiration of many EAs under anaerobic conditions [34]. Together with that Fnr is not responsive to oxygen, we intend to draw a conclusion that *S. oneidensis* Fnr plays an extremely subtle role, if not completely redundant, in anaerobic gene regulation.

It is particularly of interest to determine whether Crp mediates the aerobic-to-anaerobic respiration shift, the role that *E. coli* Fnr plays predominantly (Fig. 6). Crp differs from *E. coli* Fnr in three main aspects. First, Crp is not expected to be oxygen-sensitive as Crp in *E. coli* mainly functions under aerobic conditions. Indeed, unlike *E. coli* Fnr, *S. oneidensis* Crp functions when oxygen is abundant based on that the Crp-dependent nitrate reduction occurs in fast growing cells under aerobic conditions. Second, Crp appears to be an activator only whereas *E. coli* Fnr functions as either activator or repressor. The last but not the least, Crp is not expected to directly sense changes in oxygen concentrations as it lacks redox-sensing domains. Crp is activated by binding to cAMP, whose cellular levels depends on its synthesis by adenylyl cyclases [31]. Given that neither of these proteins is capable of sensing redox status, identifying the factors that sense redox signal and regulate levels of cellular cAMP represents an important challenge for future work.

## Methods

### Bacterial Strains, Plasmids, PCR Primers, and Culture Conditions

A list of all bacterial strains and plasmids used in this study is given in Table 2. *Escherichia coli* and *S. oneidensis* strains were grown in Luria-Bertani (LB, Difco, Detroit, MI) medium at 37°C and 30°C for genetic manipulation, respectively. Where needed, antibiotics were added at the following concentrations: ampicillin at 50 µg/ml, kanamycin at 50 µg/ml, and gentamycin at 15 µg/ml. Primers used for generating PCR products are listed in Table S1 unless otherwise noted.

**Table 2:** Strains and plasmids used in this study.

Strain or plasmid	Description	Reference or source
*E. coli* strain		
DH5α	Host for regular cloning	Lab stock
BL21(DE3)	Expression host for pTP247	[37]
WM3064	Donor strain for conjugation; Δ*dapA*	[43]
*S. oneidensis* strains		
MR-1	Wild-type	Lab stock
HG0624	*crp* deletion mutant derived from MR-1; Δ*crp*	[34]
HG0845	*napB* deletion mutant derived from MR-1; Δ*napB*	[4]
HG0848	*napA* deletion mutant derived from MR-1; Δ*napA*	[4]
HG1860	*SO1860* deletion mutant derived from MR-1; Δ*SO1860*	This study
HG3980	*nrfA* deletion mutant derived from MR-1; Δ*nrfA*	[4]
HG3982	*narP* deletion mutant derived from MR-1; Δ*narP*	This study
HG4591	*cymA* deletion mutant derived from MR-1; Δ*cymA*	[4]
Plasmids		
pDS3.0	Ap^r^, Gm^r^, derivative from suicide vector pCVD442	Lab stock
pHG101	Promoterless broad-host Km^r^ vector	[35]
pHG102	pHG101 containing the *S. oneidensis arcA* promoter	[35]
pTP247	Gateway destination His-tag expression vector	[37]
pTP247-NarP	pTP247 containing *narP*	[37]
pTP247-NarQ	pTP247 containing *narQ^51–585^*	This study
pTP247-SO1860	pTP247 containing *SO1860*	This study
pTP327	*lacZ* reporter vector	[34]
pTP327-P*_nap_*	pTP327 containing the *S. oneidensis nap* promoter	This study
pTP327-P*_nrfA_*	pTP327 containing the *S. oneidensis nrfA* promoter	This study
pTP327-P*_narQP_*	pTP327 containing the *S. oneidensis narQP* promoter	This study

### Physiological Characterization of the Mutation Strains

Batch growth under aerobic and anaerobic was assayed as described elsewhere [4], [33]. In brief, M1 defined medium containing 0.02% (w/v) of vitamin-free Casamino Acids was used unless otherwise noted. For aerobic batch growth, exponential phase cultures were diluted to approximately ∼1×10^5^ cells/ml in fresh medium and a volume of 400 µl was added into each well of plates in a Bioscreen C growth monitoring instrument (Labsystems oy, Helsinki, Finland). The cultures were shaken at medium intensity continuously. For anaerobic growth, 20 mM lactate as electron donor and as electron acceptors one of following chemicals, NaNO_3_ (5 mM), NaNO_2_ (5 mM) were supplemented. Growth of *S. oneidensis* strains was determined by monitoring an increase in OD_600_ in triplicate samples. For biochemical analyses, cells were grown in 30 ml of media supplemented with NaNO_3_/NaNO_2_ (5 mM), collected by centrifugation, frozen immediately in liquid-nitrogen, stored in −80°C for qRT-PCR, β-Galactosidase activity assay, and Western blotting and supernatants were directly used for nitrate/nitrite assays.

For cultivation under continuous culture (chemostat) conditions, cells were grown in a 1-liter working volume Bioflow III fermenter (New Brunswick Scientific, Edison, NJ, USA). To start growth, the culture in chemostats was inoculated with 1 ml of an exponential-phase batch culture of the appropriate strain grown aerobically. Cells were grown with oxygen at 20% dissolved saturation using a combination of air and N_2_ gas with parameters: temperature, 30°C; pH, 7.0±0.05; dilution rate, 0.15 h^−1^; agitation, 400 rpm. The aerobic steady phase was acquired after growth continued for at least 5 culture volumes. Suboxic and anaerobic conditions were obtained and maintained by replacing the air with the gas containing 2% oxygen and sparging the medium reservoir and the fermentor with pure nitrogen gas, respectively. The cultures were sampled by transferring the desired volume with a sterile syringe to 50 ml tubes and processed the same as batch cultures described above.

### Mutagenesis and Complementation of Mutation Strains

In-frame deletion strains were constructed using the Fusion PCR method essentially the same as previously described [33]. In brief, two fragments flanking the targeted gene were amplified independently first and joined together by the second round of PCR. The resulting fusion fragment for each individual gene was introduced into plasmid pDS3.0. The resulting mutagenesis vector was transformed into *E. coli* WM3064 [43], and then transferred into *S. oneidensis* by conjugation. Integration of the mutagenesis construct into the chromosome was selected by gentamycin resistance and confirmed by PCR. Verified transconjugants were grown in LB broth in the absence of NaCl and plated on LB supplemented with 10% of sucrose. Gentamycin-sensitive and sucrose-resistant colonies were screened by PCR for deletion of the targeted gene. The deletion mutation was then verified by sequencing of the mutated region. For complementation of genes next to their promoter, a fragment containing the targeted gene and its native promoter was generated by PCR and cloned into pHG101 [35]. For other genes, the targeted gene was amplified and inserted into MCS of pHG102 under the control of the *arcA* promoter. Introduction of each verified complementation vector into the corresponding mutant was done by mating with *E. coli* WM3064 containing the vector, and confirmed by plasmid extraction and restriction enzyme mapping. Cloned gene of interest within these two vectors is over-expressed in *S. oneidensis* and used as the positive control in western blotting.

### Expression and Purification of S. Oneidensis NarP, NarQ^51–585^ and SO1860 Proteins

The cloning of *narP* and *SO1860* has been described previously [38]. *narQ* ^51–585^ encoding a truncated NarQ lacking the transmembrane N-terminal 50 amino acids was cloned into the same system. Site-directed mutagenesis of NarP (D57N) was performed directly on the corresponding expression plasmid using a QuikChange II XL site-directed mutagenesis kit (Stratagene). Expression of the NarP, NarP (D54N), NarQ^51–585^, and SO1860 proteins in *E. coli* BL21(DE3) Star cells was induced with 0.5 mM IPTG from mid-log phase (OD_600_ = 0.5–0.6) at 30°C. The cells were grown to saturation and collected by centrifugation, resuspended in lysis buffer (50 mM Tris/HCl, pH 7.5, 200 mM NaCl, 1 mM MgCl_2_, 10 mM β-mercaptoethanol, 1 mM PMSF, 5 µg/mL DNase I), and broken by passage twice through a French press (10,000 psi). The resulting inclusion body pellets were solubilized with 20 mM Tris/HCl (pH 8.0), 5 M urea and 100 mM NaCl, and the NarP, NarP (D57N), and SO1860 proteins were further purified using Talon resin columns (BD Biosciences®) under denaturing conditions according to manufacturer’s instructions. To renature the proteins, the eluted fractions containing the purified proteins were collected, diluted into 0.8 M urea, 20 mM Tris/HCl (pH 8.0), 1 mM EDTA by sequential dilutions, and then dialyzed against 20 mM Tris/HCl (pH 7.5). The soluble NarQ^51–585^ protein was purified using a talon resin column according to manufacturer’s instructions.

### Phosphorylation and Trans-phosphorylation of NarP and SO1860

Phosphorylation of NarP and SO1860 protein was performed in buffer containing 100 mM Tris/HCl (pH 7.0), 10 mM MgCl_2_, 125 mM KCl, 50 mM dilithium carbamoyl phosphate for 60 minutes at room temperature as described [33]. The trans-phosphorylation of NarP and SO1860 by NarQ^51–585^ was performed in 20 µl reaction volume as previously described [33], with the modification that 0.1 mM [γ-^33^P]ATP was used instead of [γ-^32^P]ATP and the non-radioactive ATP was used at 1 mM when indicated.

### Electrophoretic Motility Shift Assay (EMSA)

The probes used for EMSA were prepared by PCR amplification with ^33^P end-labeled primers. The binding reaction was performed with ∼25 fmol (∼2 nM) labeled probes and various amount of protein in 12 µl binding buffer containing 100 mM Tris/HCl (pH 7.4), 20 mM KCl, 10 mM MgCl_2_, 2 mM DTT, and 10% glycerol at 15°C for 60 minutes and resolved on pre-run 4.8% polyacrylamide native gels [33]. The DNA bands were visualized by autoradiography.

### Construction of Transcriptional Fusion and β-Galactosidase Activity Assay

To determine the activity of the target *S. oneidensis* promoters, sequences of target promoters were amplified and cloned into transcriptional fusion vector pTP327 using the restriction sites within primers listed in Table S1 [34]. The resulting transcriptional fusion vector was transformed into *E. coli* WM3064, verified by sequencing, and transferred into *S. oneidensis* strains by conjugation. β-Galactosidase activity assay was performed using an assay kit (Beyotime, China) according to manufacturer’s instructions as described previously [35]. The activity was expressed in Miller units as described by Miller [44].

### Quantitative RT-PCR (qRT-PCR) Analysis

Quantitative real-time reverse transcription-PCR (qRT-PCR) analysis was carried out with a Mastercycler 96-well qRT-PCR system (Eppendorf) essentially the same as described previously [45]. The expression of each gene was determined from three replicates on a single real-time qRT-PCR experiment. The Cycle threshold (*C_T_*) values for each gene of interest were averaged and normalized against the *C_T_* value of 16s rRNA, whose abundance was consistent from early exponential phase to stationary phase. The relative abundance (RA) of each gene compared to that of 16s rRNA was calculated using the equation RA = 2^−Δ*CT*^.

### Immunoblotting Assay

Rabbit polyclonal antibodies against fragments of NrfA (CFTDHKVGNPFDRFE), Crp (LIGKPKPDPTLEWFC), and NarP (CKDTEPDLLLDKLKN) were prepared in accordance with standard protocols provided by the manufacturer (Genscript) and used for the immunoblotting analysis [35]. Cell pellets were washed once with PBS, and resuspended to an optical density at 600 nm (OD_600_) of PBS. The total protein concentration of the cell lysates was then determined by the bicinchoninic acid assay (Pierce Chemical). Samples were loaded onto SDS-10% polyacryl-amide gels and either stained with Coomassie brilliant blue or electrophoretically transferred to polyvinylidene difluoride (PVDF) according to the manufacturer’s instructions (Bio-Rad). The gels were blotted for 2 h at 60 V using a Criterion blotter (Bio-Rad). The blotting membrane was probed with anti-NrfA antibody, anti-NarP antibody followed by a 1∶5,000 dilution of goat anti-rabbit IgG-HRP (Horse radish peroxidase) (Roche Diagnostics) was detected using a chemiluminescence Western blotting kit (Roche Diagnostics) in accordance with the manufacturer’s instructions. Images were visualized with the UVP Imaging System.

### Chemical Assays

Culture supernatants were subjected to Ion Chromatography (IC) analysis for determination of nitrate and nitrite concentrations essentially the same as previously described [4]. The assay was performed with IonPac^®^ AS19 and Na_2_SO_4_ as the eluent at a concentration of 100 mM with a flow rate of 0.6 ml/min in ICS-3000 (Dionex, Sunnyvale, CA, USA). Nitrite quantification was also carried out according to the method by Miranda et al. [46]. Standard curve was made each time.

## Supporting Information

Figure S1 **Physiological characterization of mutant strains.** The experiments were performed at least three times. In all panels, error bars (Standard deviation <5%) were omitted for clarification. **A.** Aerobic growth of *S. oneidensis* strains in the presence of 5 mM NaNO_3_ (solid line) or NaNO_2_ (dash line). Mutants used here have been previously confirmed by genetic complementation [4]. **B.** Nitrite reduction of *S. oneidensis* Δ*cymA*. 5 mM nitrite was initially added. Δ*cymA^c^* represents the Δ*cymA* strain containing a copy of *cymA* with its own promoter on pHG101. The Δ*napA* and Δ*napA^c^* strains were included as the control. **C.** Growth of *S. oneidensis* Δ*narP* and Δ*SO1860* in the presence of 5 mM nitrate under anaerobic conditions. Δ*narP^c^* represents the Δ*narP* strain containing a copy of *narP* under the control of P*_arcA_* within pHG102 [35].
(PDF)

Figure S2 **Sequence comparison of the** ***S. oneidensis*** **and** ***E. coli*** **NarP proteins.** The predicted phosphorylation residues, Asp57 in *S. oneidensis* NarP and Asp59 in *E. coli* NarP, were marked.
(PDF)

Figure S3 **Expression of** ***napA*** **,** ***narfA*** **,** ***narP*** **in** ***S. oneidensis*** **strains.** WT/*napA* represents expression of *napA* in WT strain. **A.** *lacZ*-based reporter analysis of the *nap* and *nrfA* promoters in Δ*narP*. Expression of *nap* and *nrfA* in the wild type and Δ*narP* cells cultured aerobically in the presence of nitrate and nitrite, respectively, were shown. **B.** qRT-PCR analysis of *napA, nrfA,* and *narP* in the wild-type, Δ*crp*, and Δ*fnr* cells grown with 5 mM nitrite aerobically. Abundance is given relative to 16 S rRNA.
(PDF)

Table S1 **Primers used in this study.**
(PDF)
