# Supplementary material for: A Crp-Dependent Two-Component System Regulates Nitrate and Nitrite Respiration in Shewanella oneidensis
Source: PLoS One. 2012 Dec 11;7(12):e51643. doi: 10.1371/journal.pone.0051643 (PMC3519889; doi:10.1371/journal.pone.0051643)
Supplement: Figure S1 — Physiological characterization of mutant strains. The experiments were performed at least three times. In all panels, error bars (Standard deviation <5%) were omitted for clarification. A. Aerobic growth of S. oneidensis strains in the presence of 5 mM NaNO3 (solid line) or NaNO2 (dash line). Mutants used here have been previously confirmed by genetic complementation [4]. B. Nitrite reduction of S. oneidensis ΔcymA. 5 mM nitrite was initially added. ΔcymAc represents the ΔcymA strain containing a copy of cymA with its own promoter on pHG101. The ΔnapA and ΔnapAc strains were included as the control. C. Growth of S. oneidensis ΔnarP and ΔSO1860 in the presence of 5 mM nitrate under anaerobic conditions. ΔnarPc represents the ΔnarP strain containing a copy of narP under the control of ParcA within pHG102 [35]. (PDF) [file pone.0051643.s001.pdf]

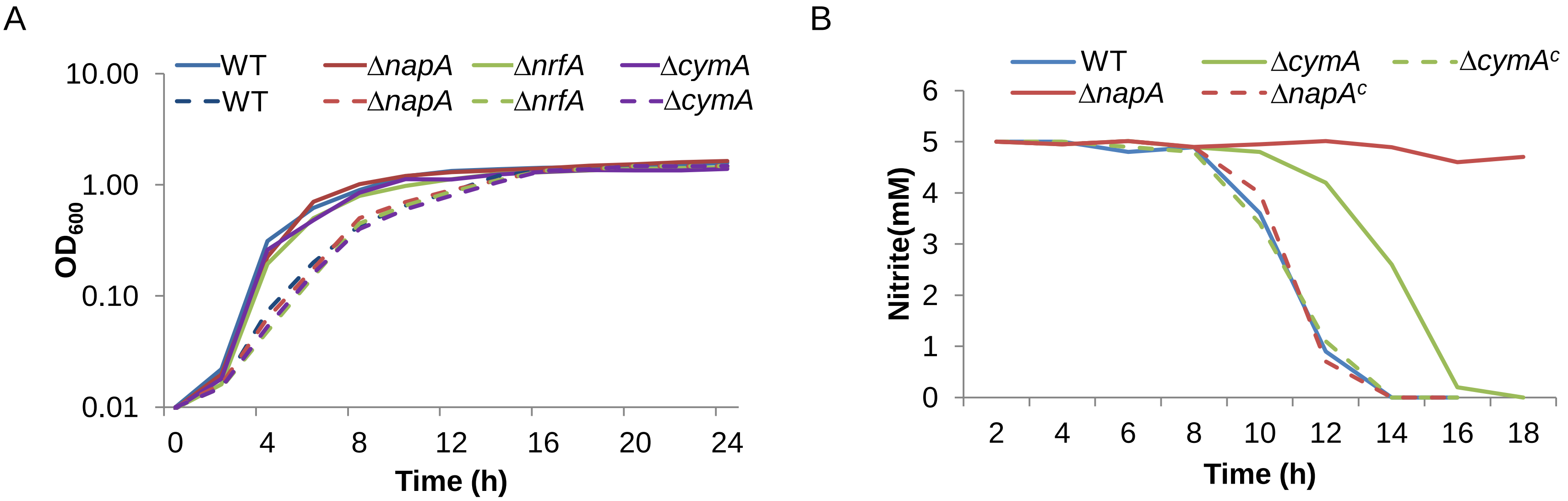

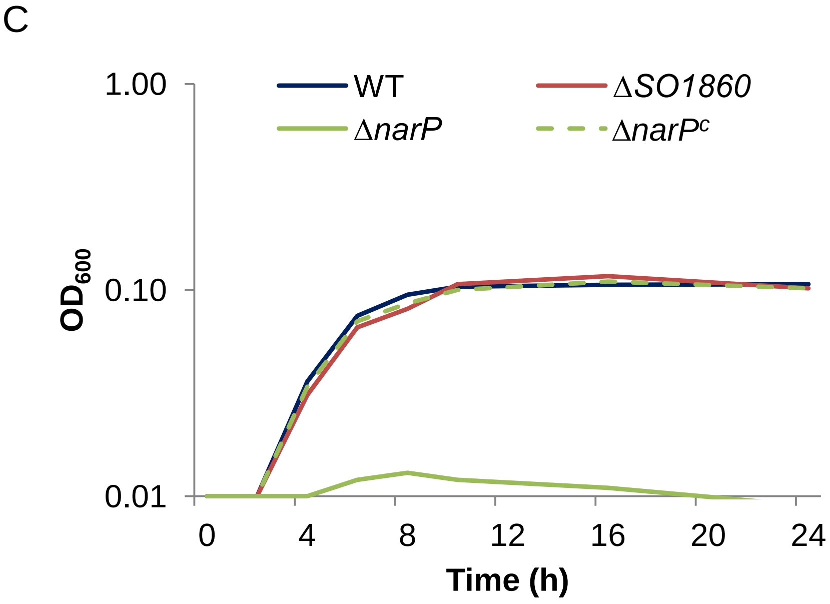

Figure S1. Physiological characterization of mutant strains. The experiments were performed at least three times. In all panels, error bars (Standard deviation < 5%) were omitted for clarification.

A. Aerobic growth of *S. oneidensis* strains in the presence of 5 mM $NaNO_3$ (solid line) or $NaNO_2$ (dash line). Mutants used here have been previously confirmed by genetic complementation [4].

B. Nitrite reduction of *S. oneidensis* Δ*cymA*. 5 mM nitrite was initially added. Δ*cymA*^{c} represents the Δ*cymA* strain containing a copy of *cymA* with its own promoter on pHG101. The Δ*napA* and Δ*napA*^{c} strains were included as the control.

C. Growth of *S. oneidensis* Δ*narP* and Δ*SO1860* in the presence of 5 mM nitrate under anaerobic conditions. Δ*narP*^{c} represents the Δ*narP* strain containing a copy of *narP* under the control of P_{arcA} within pHG102 [35].
