# Supplementary material for: A Crp-Dependent Two-Component System Regulates Nitrate and Nitrite Respiration in Shewanella oneidensis
Source: PLoS One. 2012 Dec 11;7(12):e51643. doi: 10.1371/journal.pone.0051643 (PMC3519889; doi:10.1371/journal.pone.0051643)
Supplement: Figure S2 — Sequence comparison of the S. oneidensis and E. coli NarP proteins. The predicted phosphorylation residues, Asp57 in S. oneidensis NarP and Asp59 in E. coli NarP, were marked. (PDF) [file pone.0051643.s002.pdf]

```
SoNarP      --MGKPYSVLVVDDHPLLRKGICQLITSDPDFSLFGEVGSGLDALSSVATDEPDIVLLDL 58
EcNarP      MPEATPFQVMIVDDHPLMRRGVRQLLELDPGSEVVAEAGDGASAIDLANRLDIDVILLDL 60
               ..*:.*::******:*:*: **:  **. .:..*.*.* .*:. .   : *::****

SoNarP      NMKGMTGLDTLNAMRQEGVTSRIVILTVSDAKQDVIRLLRAGADGYLLKDTEPDLLLDKL 118
EcNarP      NMKGMSGLDTLNALRRDGVTAQIIILTVSDASSDVFALIDAGADGYLLKDSDPEVLLEAI 120
            *****:*******:*::***::*:*******..**: *: **********::*::**: :

SoNarP      KNTMSGHRVISEEVAEYLYELKNAADEQEWVSSLTPRELQILQQLAEGLSNRMISEHLHI 178
EcNarP      RAGAKGSKVFSERVNQYLREREMFGAEEDPFSVLTERELDVLHELAQGLSNKQIASVLNI 180
            :   .* :*:**.* :** * :  . *:: .* ** ***::*::**:****: *:. *:*

SoNarP      SEGTVKVHVKNLLRKANAKSRTEMAVRYLNN---- 209
EcNarP      SEQTVKVHIRNLLRKLNVRSRVAATILFLQQRGAQ 215
            ** *****::***** *.:**.  :: :*::
```

Figure S2. Sequence comparison of the *S. oneidensis* and *E. coli* NarP proteins. The predicted phosphorylation sites, Asp57 in *S. oneidensis* NarP and Asp59 in *E. coli* NarP, were marked.
