# Supplementary material for: A Crp-Dependent Two-Component System Regulates Nitrate and Nitrite Respiration in Shewanella oneidensis
Source: PLoS One. 2012 Dec 11;7(12):e51643. doi: 10.1371/journal.pone.0051643 (PMC3519889; doi:10.1371/journal.pone.0051643)
Supplement: Figure S3 — Expression of napA , narfA , narP in S. oneidensis strains. WT/napA represents expression of napA in WT strain. A. lacZ-based reporter analysis of the nap and nrfA promoters in ΔnarP. Expression of nap and nrfA in the wild type and ΔnarP cells cultured aerobically in the presence of nitrate and nitrite, respectively, were shown. B. qRT-PCR analysis of napA, nrfA, and narP in the wild-type, Δcrp, and Δfnr cells grown with 5 mM nitrite aerobically. Abundance is given relative to 16 S rRNA. (PDF) [file pone.0051643.s003.pdf]

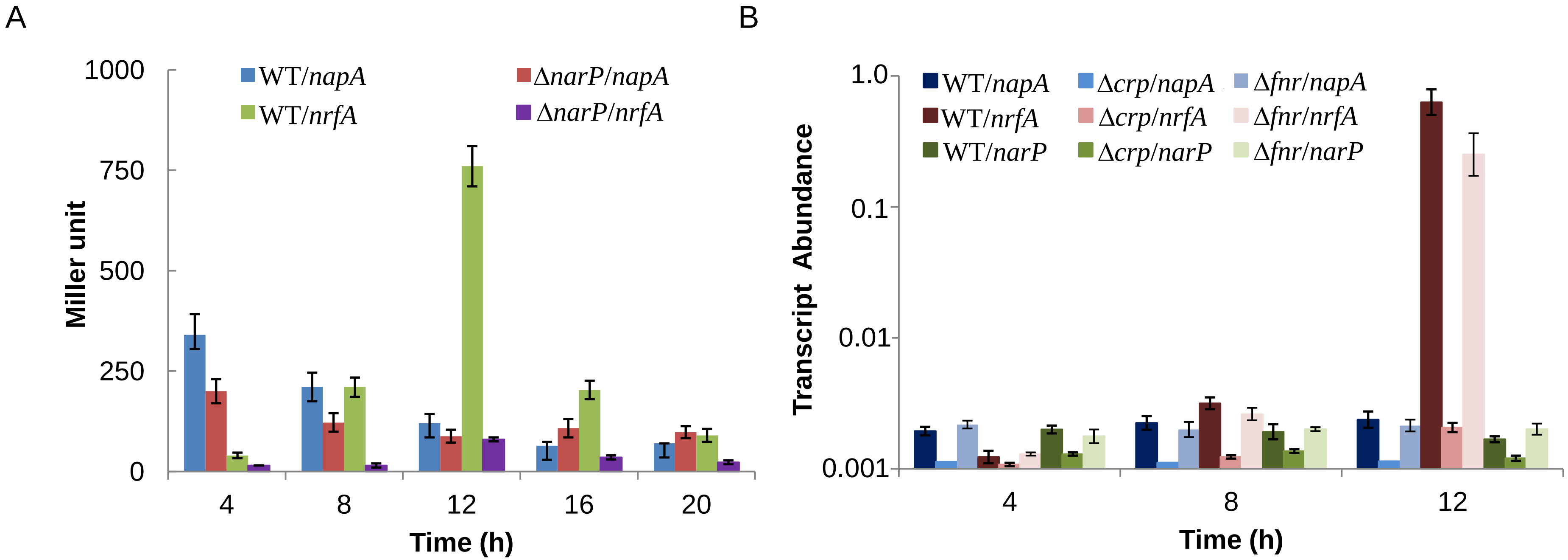


**Figure S3.** Expression of *napA*, *narfA*, *narP* in *S. oneidensis* strains. WT/*napA* represents expression of *napA* in WT strain.
A. *lacZ*-based reporter analysis of the *nap* and *nrfA* promoters in Δ*narP*. Expression of *nap* and *nrfA* in the wild type and Δ*narP* cells cultured aerobically in the presence of nitrate and nitrite, respectively, were shown.
B. qRT-PCR analysis of *napA*, *nrfA*, and *narP* in the wild-type, Δ*crp*, and Δ*fnr* cells grown with 5 mM nitrite aerobically. Abundance is given relative to 16S rRNA.
