# Supplementary material for: A Crp-Dependent Two-Component System Regulates Nitrate and Nitrite Respiration in Shewanella oneidensis
Source: PLoS One. 2012 Dec 11;7(12):e51643. doi: 10.1371/journal.pone.0051643 (PMC3519889; doi:10.1371/journal.pone.0051643)
Supplement: Table S1 — Primers used in this study. (PDF) [file pone.0051643.s004.pdf]

Table S1. Primers used in this study

Mutagenesis	
SO1860-5-F	GGTTTACAGTCTTGGGCGA
SO1860-5-R	CTAAGTAGCCATGACGACGATCGATATATCAACTTTATCCACC
SO1860-3-F	CGTCGTCATGGCTACTTAGTCGTTTAGCGATCCGCTATAA
SO1860-3-R	CCGCATTACTGACTTTACCAAC
SO3982-5-F	GTACAGCTAAGGGAGT
SO3982-5-R	TGTTTAAACTTAGTGGATGGGGTGGTCGTCCACCA
SO3982-3-F	CCCATCCACTAAGTTTAAACACCGAGATGGCGGTA
SO3982-3-R	CAGTTTGACAGGCACG
Complementation	
SO3980-C1-F	AActgcagTAACAACGCGGAGATGAGA
SO3980-C1-R	CCGgaattcCGTCCAAGTAGTTCAACAAA
SO3980-C2-F	CGCggatccAAGCGTTGATGGCTTTC
SO3980-C2-R	CCGgaattcCGTCCAAGTAGTTCAACAAA
SO3982-C-F	AActgcagATGGGTAAACCTTATTCAGTT
SO3982-C-R	CCGgaattcTCAGCAAGTGCTCGATAT
SO0624-C-F	CGCggatccCCATCAGGTCACTAAGGTTT
SO0624-C-R	CCGgaattcCCACTTTAATGATACAGGCTTA
qRT-PCR	
SO0624-q-F	ATGATCCTATCTTATCTTAA
SO0624-q-R	GCAATTTCGCATGCCTGTTT
SO0970-q-F	GGCCAATGTGTTAGCTGCCA
SO0970-q-R	CTCGTACAAGCGATTTCACC
SO3980-q-F	GCCAAAGATTATAAAGCCCCTCG
SO3980-q-R	AGGCACGTCAGGACTTTTACAGC
SO3982-q-F	GATGCCTTAAGCAGCGTTGC
SO3982-q-R	TCGTGAAGTCACGCCTTCTT
16srDNA-q-F	AAGAAGGACCGGCTAACTCC
16srDNA-q-R	TTCACATCTCGCTTAACAAACC
LacZ Reporter System	
PnrfA-F	CCGgaattcATCCGCAGTGAACCCGAA
PnrfA-R	CGCggatccGTAATCATGGTCATGGCAACCAATGCACTTAATGC
SO3980-R-F	CCGgaattcATCCGCAGTGAACCCGAA
SO3980-R-R	CGCggatccGTAATCATGGTCATGGCAACCAATGCACTTAATGC
SO0848-R-F	CCGgaattcCCACACAGGCATCCCACT
SO0848-R-R	CGCggatccgtaatcatggTCATTTCTTGTTCTAGGGGCTCGAC
EMSA	
NAP-E-F	AAGGGATAGCGATTGTTTAA
NAP-E-R	GTACAACTAAATGTGGATAC
NRFA-E-F	AGCCGTGTTTTTTACATTAG
NRFA-E-R	AAGTTAGTAGCTAAATACCG
